# Effect of Dietary Conjugated Linoleic Acid Supplementation on Early Inflammatory Responses during Cutaneous Wound Healing

**DOI:** 10.1155/2010/342328

**Published:** 2010-08-17

**Authors:** Na-Young Park, Giuseppe Valacchi, Yunsook Lim

**Affiliations:** Department of Food and Nutrition, Research Institute of Science for Human Life, Kyung Hee University, 1 Hoegi-dong, Dongdaemun-gu, Seoul 130-761, Republic of Korea

## Abstract

Inflammatory response is considered the most important period that regulates the entire healing process. Conjugated linoleic acid (CLA), a class of linoleic acid positional and geometric isomers, is well known for its antioxidant and anti-inflammatory properties. We hypothesized that dietary CLA supplementation accelerates cutaneous wound healing by regulating antioxidant and anti-inflammatory functions. To investigate wound closure rates and inflammatory responses, we used a full-thickness excisional wound model after 2-week treatments with control, 0.5%, or 1% CLA-supplemented diet. Mice fed dietary CLA supplementation had reduced levels of oxidative stress and inflammatory markers. Moreover, the wound closure rate was improved significantly in mice fed a 1% CLA-supplemented diet during early stage of wound healing (inflammatory stage). We conclude that dietary CLA supplementation enhances the early stage of cutaneous wound healing as a result of modulating oxidative stress and inflammatory responses.

## 1. Introduction

Wound healing is an essential procedure that helps maintain homeostasis and integrate tissue injured by physical, chemical, bacterial, or viral insults [1–3]. Generally, there are three major stages of wound healing that overlap in time and space: inflammation, proliferation, and remodeling [4]. Because it is involved in producing growth factors and cytokines that coordinate the cell and tissue movements necessary for repair, the inflammatory response is considered the most important period that regulates the entire healing process [5–7]. The initial event of the inflammatory stage during wound healing is the infiltration of neutrophils and macrophages into the wound site to attack contaminating bacteria and to phagocytose cellular debris resulting in the production of reactive oxygen species (ROS). Furthermore, neutrophils and macrophages are both major sources and targets of proinflammatory cytokines such as IL-1*α*, IL-1*β*, IL-6, and TNF-*α* that have been shown to be crucial mediators during cutaneous inflammatory processes [4, 5]. For example, IL-1*β* and TNF-*α*, primary proinflammatory cytokines, promote nuclear factor *κ*B (NF*κ*B) activation and ROS production in inflammatory cells [8–10]. NF*κ*B is a redox-sensitive transcription factor that acts as a central protein regulating the transcription of many inflammatory mediators including cyclooxygenase (COX)-2, a rate-limiting enzyme in the biosynthesis of prostaglandins during inflammation and immune response. Because of its essential role in controlling inflammatory responses, regulation of NF*κ*B activation by the oxidative stress present during the early inflammatory cascade may be advantageous in the treatment of wounds.

Cells in injured and inflamed tissues are able to protect themselves with a well-equipped array of antioxidant enzymes, such as glutathione peroxidase (GPX), catalase, superoxide dismutases (SODs), and heme oxygenases (HO). SODs dismutate superoxide radical anions to H_2_O_2_ and water. A previous study has shown that mRNA levels of CuZnSOD and MnSOD were upregulated in the early inflammatory stage of cutaneous wound healing [11]. HO-1, the inducible isoform of HO, shows protection against ROS as well. In mouse full-thickness excisional wounds, the mRNA and protein levels of HO-1 increased after wounding and subsequently declined until the wound was healed [11], which proposes a role of HO-1 in a protective function through reduction of inflammation [12].

Despite the remarkable significance of inflammation, most studies have focused on the later stage of wound healing, the remodeling stage, especially in collagen formation and maturation [13]. Our group previously demonstrated that N-acetylcysteine and zinc known as antioxidant nutrients played important role in regulation of wound healing rate and inflammatory response during wound healing procedure [1, 14]. However, except these two antioxidant nutrients, little is known on the molecular mechanism that controls the wound healing rate. 

Conjugated linoleic acid (CLA) refers to a class of the essential fatty acid linoleic acid positional and geometric isomers, marked by conjugated double bond [15]. CLA was found naturally in meat and dairy products from cows and sheep due to the process of bacterial biohydrogenation of linoleic acid (LA) in the rumen [16]. Interest in CLA came first from its anticarcinogenic action [17] and now there is a growing literature showing the beneficial effects of CLA, such as antiadipogenic [15, 18], antiatherogenic [19, 20], antidiabetogenic [21, 22], anti-inflammatory [23–26], and antioxidant properties [27–29]. It was suggested that CLA is effective in improving skin disorders in animals [30]. In addition, Hwang et al. have reported that CLA inhibited NF*κ*B-driven COX-2 protein expression in mouse skin carcinogenesis [31]. However, the effect of CLA on cutaneous wound healing is poorly documented. 

We hypothesized that dietary CLA supplementation promotes cutaneous wound healing through modulation of inflammatory responses including NF*κ*B activation. In particular, we used a cutaneous full-thickness excisional wound model to examine the effects of dietary CLA supplementation on the rate of wound closure, and the temporal expression levels of pI*κ*B*α*, COX-2, CuZnSOD, MnSOD, and HO-1 protein levels during the early inflammatory stage of cutaneous wound healing.

## 2. Materials and Methods

### 2.1. Animals and Diets

Female CrljBgi : CD-1 (ICR) mice (4 wks old) were purchased from Orient Bio Inc. (Gyeonggi-do, South Korea) and lodged in individual plastic cages at temperature- and humidity-controlled room (12-hr light:dark cycle) with allowed access to distilled water and food. Body weights and food intake were measured every three days and every day, respectively. Mice were acclimated for 7 days (d) before starting dietary treatments. They were fed a modified AIN-93G rodent diet supplemented with 0 (control), 0.5%, or 1% CLA for 2 wks (Han Live R&D, Gyeonggi-do, South Korea) (Tables 1 and 2). All mice were used in accordance with animal protocols approved by the Kyung Hee University Institutional Animal Care and Use Committee.

### 2.2. Wound Biopsy

Mice were anesthetized with isoflurane and the back of the mouse was shaved using an electronic clipper and sterilized using an alcohol swab. The wound biopsy model used in this experiment has been previously described [32]. The skin was then nipped and bent, and the full-thickness excisional wounds were made on the bent skin using a sterile biopsy punch (4 mm diameter, Kai medical, Gifu City, Japan). Two round shape wounds on the dorsum, below the shoulder blades of each mouse were made to avoid self-licking.

### 2.3. Harvesting

Mice were sacrificed with an overdose of ethyl ether to gather tissue samples at the wound site. Each wound site at 0, 24, 48, and 72 hours (hr) after wounding and at end point was harvested by a cutting area that covered the entire wound site.

### 2.4. Malondialdehyde Measurement

Levels of malondialdehyde (MDA) were measured as a marker of lipid peroxidation [33, 34]. Ten percent (weight/volume) liver tissues were homogenized with 0.15 M KCl buffer. 0.2 ml of tissue homogenate, 0.2 ml sodium dodecyl sulphate, 8.1% (weight/volume), and 3 ml 20% acetic acid-0.8% TBA mixture (1 : 1, by volume) were added, to reach the volume of 4 ml. Samples were then blended, warmed for 60 minutes (min), maintained at 95°C, and then cooled under tap water immediately. To each tube, 1 ml distilled water and 5 ml mixture of n-butanol and pyridine (15 : 1, by volume) were supplemented and shaken vigorously and centrifuged at 4,000 rpm for 10 min. The upper layer was aspirated out and absorbancy was measured at 532 nm using 1,1,3,3-tetramethoxypropane as an external criterion.

### 2.5. Measurement of Wound Closure

Wounds from each mouse were photographed digitally every day, beginning on the day of wounding (d 0) with a criterion spot equal to the initial wound part placed beside the wound. The quantification of wound closure used in this experiment was previously described [35]. The rate of wound closure was demonstrated as the ratio of the wound size (each day after wounding) compared with the initial wound size. A smaller wound ratio indicates faster wound closure.

### 2.6. Preparation of Homogenized Skin Tissue Proteins and Western Blot

Skin tissues from the wound sites were homogenized in lysis buffer (20 mM Tris-HCl (Sigma, St. Louis, MO, USA), 150 mM NaCl_2_ (Duksan, Gyeonggi-do, South Korea), pH7.5, 1% NP40 (Sigma), 0.5% Na-deoxycholate stock (Sigma), 1mM EDTA (Duksan), and 0.1% sodium dodecyl sulfate (Sigma)) as previously described by our group [36]. The homogenates were incubated on ice for 20 min and centrifuged at 14,000 rpm for 30 mins. The supernatants were stored at −80°C until additional processing.

For Western blot analysis, samples (60 *μ*g protein, determined using BioRad protein assay; BioRad, Hercules, CA, USA) were separated on 10% SDS-PAGE gels and electrotransferred onto PVDF (polyvinylidene fluoride) membranes. After blocking in 5% nonfat milk in PBS-Tween 20, the membranes were incubated with specific monoclonal and polyclonal antibodies against phosphorylated I*κ*B*α* (pI*κ*B*α*) (Santa Cruz Biotechnology, Santa Cruz CA, USA, 1 : 200), COX-2 (Transduction Laboratories, Lexington KY, USA, 1 : 250), HO-1 (Stressgen, Victoria BC, Canada, 1 : 2000), CuZnSOD (Santa Cruz Biotechnology, 1 : 500), MnSOD (Stressgen, 1 : 5000), and *β*-actin (Santa Cruz Biotechnology, 1 : 200). Horseradish peroxidase conjugated antibody (Santa Cruz Biotechnology) was used as secondary antibody and bound antibodies were visualized by enhanced chemiluminescence (ECL Western Blotting Substrate, Pierce), captured on X-ray film (Agfa-Gevaert N.V.), and band densities were quantified using NIH image software.

### 2.7. Statistical Analysis

All values are expressed as means ± SEM. Data were analyzed by 1-way ANOVA, and then differences among means were analyzed using Duncan's test. The relationships between body weight gain and wound size were evaluated by Pearson's correlation coefficients. For all tests, differences were considered significant at *P* < .05.

## 3. Results

### 3.1. Effect of Dietary CLA Supplementation on Body Weight and Food Intake

**Figure 1:**
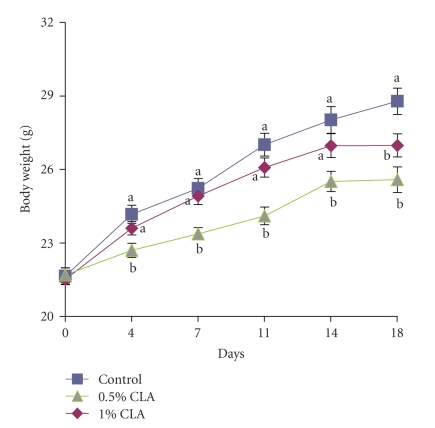
Changes in body weight in mice fed diets containing 0 (control), 0.5%, or 1% CLA for 2 weeks. Values are means ± SEM; *N* = 4 to 6 in each group. Means at a time with a different letter are significantly different, *P* < .05.

Dietary CLA supplementation significantly reduced the body weights gain in mice throughout the experimental periods compared to a control diet (Figure 1). Particularly, body weight gain was lower in the 1% CLA-supplemented group than the control and 0.5% CLA-supplemented groups (*P* < .05). The food intakes during the experiments were not significantly different among the groups (data not shown).

### 3.2. Effect of Dietary CLA Supplementation on MDA Levels of Mouse Liver

**Figure 2:**
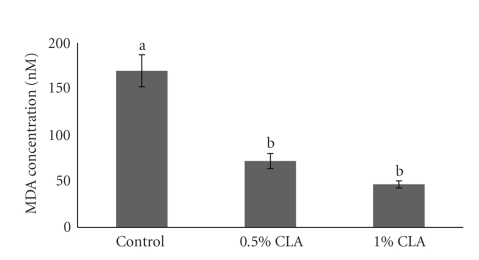
Liver MDA levels in mice fed diets containing 0 (control), 0.5%, or 1% CLA for 2 weeks. Values are means ± SEM; *N* = 4 to 6 in each group. Means with a different letter are significantly different, *P* < .05.

Dietary CLA supplementation resulted in a decrease of liver lipid peroxidation measured as MDA levels (Figure 2). Liver MDA levels were significantly lower in CLA-supplemented groups as compared to the controls (about 2.4- to 3.7-times, resp.). No differences between the 0.5% and the 1% CLA-supplemented groups were noted.

### 3.3. Effect of Dietary CLA Supplementation on Wound Closure Rate

**Figure 3:**
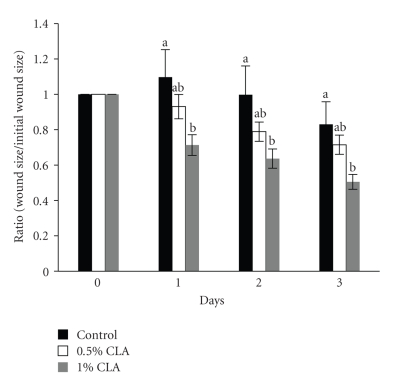
The rate of wound closure in mice fed diets containing 0 (control), 0.5%, or 1% CLA for 2 weeks. The area of the wound of each time point was relative to the area of the wound on d 0 (set at 1.0). Values are means ± SEM; *N* = 4 to 6 in each group. Means at a time with a different letter are significantly different, *P* < .05.

The rate of wound closure in the 1% CLA-supplemented group was significantly faster than those of the control and the 0.5% CLA-supplemented groups during the early stage of wound healing (Figure 3). Although not significant, the 0.5% CLA-supplemented groups showed a faster wound closure rate than the control, suggesting a dose-dependent effect of CLA.

### 3.4. Effect of Dietary CLA Supplementation on Relationships between Body Weight Gain and Early Wound Size

We performed statistical analysis to identify correlations between body weight gain and early wound size as shown in Table 3. Body weight was positively correlated to early wound size (*P* < .05).

### 3.5. Effect of Dietary CLA Supplementation on pI*κ*B*α* Expression Levels

**Figure 4:**
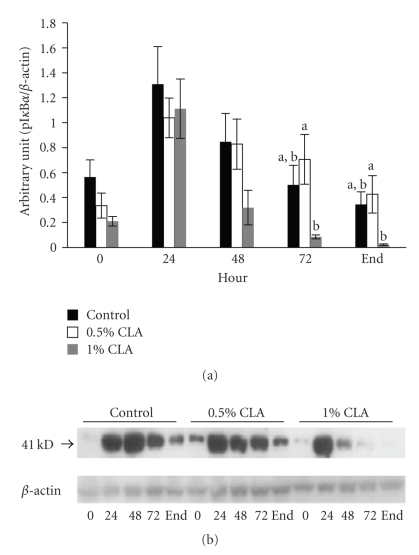
pI*κ*B*α* protein expression in cutaneous wounds of mice fed diets containing 0 (control), 0.5%, or 1% CLA for 2 weeks. (a) Quantified pI*κ*B*α* protein expression levels determined by densitometry. Values are mean ± SEM; *N* = 4 to 6 in each group. Means at a time with a different letter are significantly different, *P* < .05. (b) 0; 0 hr, 24; 24 hr, 48; 48 hr, 72; 72 hr after wounding, end (wound is completely closed).

During wound healing, pI*κ*B*α* protein level increased in all groups. The expression levels of pI*κ*B*α* in the 1% dietary CLA supplemented group were significantly reduced starting at 48 hr after wounding compared to the other groups (Figure 4).

### 3.6. Effect of Dietary CLA Supplementation on COX-2 Expression in Mouse Skin

**Figure 5:**
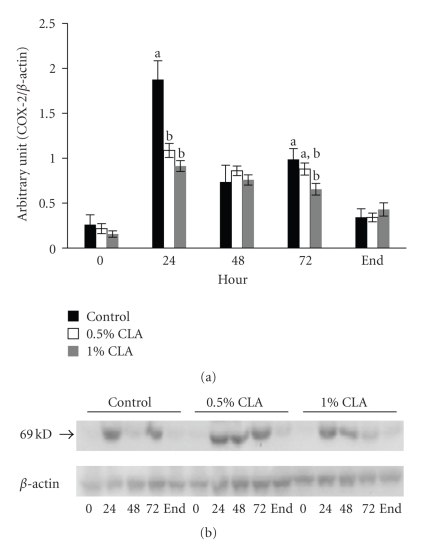
COX-2 protein expression in cutaneous wounds of mice fed diets containing 0 (control), 0.5%, or 1% CLA for 2 weeks. (a) Quantified COX-2 protein expression levels determined by densitometry. Values are mean ± SEM; *N* = 4 to 6 in each group. Means at a time with a different letter are significantly different, *P* < .05. (b) 0; 0 hr, 24; 24 hr, 48; 48 hr, 72; 72 hr after wounding, end (wound is completely closed).

As shown in Figure 5, the expression of COX-2 protein was barely detectable at baseline (0 hr), and it was induced in all groups after wounding. The expression levels of COX-2 at 24 hr after wounding were remarkably reduced in the dietary CLA-supplemented mice (*P* < .05) and returned to the steady levels in all groups at the end point.

### 3.7. Effect of Dietary CLA Supplementation on HO-1 Expression in Mouse Skin

**Figure 6:**
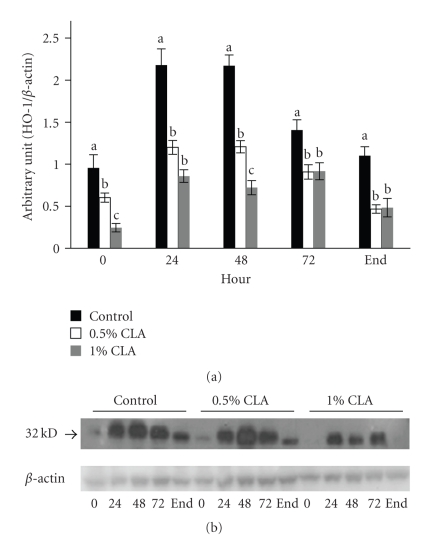
HO-1 protein expression in cutaneous wounds of mice fed diets containing 0 (control), 0.5%, or 1% CLA for 2 weeks. (a) Quantified HO-1 protein expression level determined by densitometry. Values are mean ± SEM; *N* = 4 to 6 in each group. Means at a time with a different letter are significantly different, *P* < .05. (b) 0; 0 hr, 24; 24 hr, 48; 48 hr, 72; 72 hr after wounding, end (wound is completely closed).

Significant decreases in HO-1 protein levels in the dietary CLA-supplemented groups were shown at all time points compared with the control group during wound healing (Figure 6).

### 3.8. Effect of Dietary CLA Supplementation on CuZnSOD Expression in Mouse Skin

**Figure 7:**
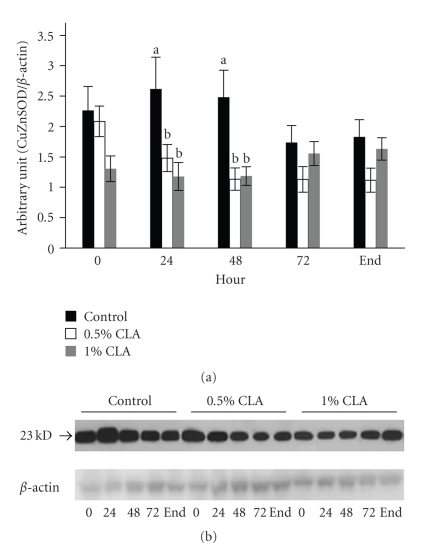
CuZnSOD protein expression in cutaneous wounds of mice fed diets containing 0 (control), 0.5%, or 1% CLA for 2 weeks. (a) Quantified CuZnSOD protein expression level determined by densitometry. Values are mean ± SEM; *N* = 4 to 6 in each group. Means at a time with a different letter are significantly different, *P* < .05. (b) 0; 0 hr, 24; 24 hr, 48; 48 hr, 72; 72 hr after wounding, end (wound is completely closed).

Expression levels of CuZnSOD protein in the control group were significantly higher at 24 hr and 48 hr after wounding (2 folds) as compared with those in the dietary CLA-supplemented groups (Figure 7).

### 3.9. Effect of Dietary CLA Supplementation on MnSOD Expression in Mouse Skin

**Figure 8:**
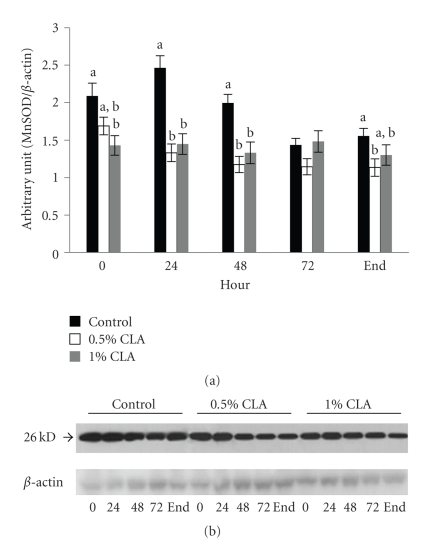
MnSOD protein expression in cutaneous wounds of mice fed diets containing 0 (control), 0.5%, or 1% CLA for 2 weeks. (a) Quantified MnSOD protein expression level determined by densitometry. Values are mean ± SEM; *N* = 4 to 6 in each group. Means at a time with a different letter are significantly different, *P* < .05. (b) 0; 0 hr, 24; 24 hr, 48; 48 hr, 72; 72 hr after wounding, end (wound is completely closed).

Like CuZnSOD, we also detected a significant increase in MnSOD protein in the controls compared to the dietary CLA-supplemented groups at 24 hr and 48 hr after wounding (Figure 8). MnSOD protein levels in the controls were clearly increased after wounding, while no changes were noted in the dietary CLA-supplemented groups.

## 4. Discussion

In these experiments, we investigated the effects of dietary CLA supplementation on molecular events in the early inflammatory stage of cutaneous wound healing using an *in vivo *model. Our data demonstrated that dietary CLA supplementation decreased weight gain, reduced the levels of both inflammatory and oxidative stress markers at wound sites, and accelerated the wound closure rate.

We found weight loss effects of dietary CLA supplementation in mice as confirmed by previous studies. The effects of CLA in regulating body weight have been supported by several animal studies [15, 18]. Reduction in body weight seems to be mainly due to the decreased adipose deposition, as a consequence of a gain in lean mass or protein substances. Moreover, the present study supported a correlation between obesity and increased morbidity and mortality after traumatic injury. Previous studies showed that obese trauma patients have a higher likelihood to experience postoperative infectious complications, incidence of multiple organ dysfunction, and longer hospitalization when compared with nonobese patients after acute injury [37–39]. Our date are in accordance with the above-mentioned reports since a lower body weight gain by dietary CLA supplementation improved the wound closure rate.

Previous studies showed that dietary CLA enhanced oxidative stability of liver [40, 41], which suggested that dietary CLA supplementation increases the ability of protection to oxidative stress and scavenging free radicals and this can explain the decreased MDA levels, the marker of lipid peroxidation, observed in liver of dietary CLA supplemented mice (Figure 2). 

To understand part of the molecular mechanisms through which dietary CLA supplementation modifies the rate of the wound closure, we investigated NF*κ*B activation through the expression of pI*κ*B*α*. NF*κ*B is sequestered in the cytoplasm of unstimulated cells bound to the inhibitory protein, I*κ*B [42]. Exposure of cells to various stimuli such as ROS, proinflammatory cytokines, UV light, or bacterial endotoxins results in NF*κ*B activation and then I*κ*B is phosphorylated and degraded, promoting the translocation of NF*κ*B to the nucleus where it controls the expression of specific cellular genes associated with host inflammatory and immune response [42]. Our data demonstrated that pI*κ*B*α* protein levels generally were downregulated in mice fed dietary CLA supplementation than in mice fed the control diet (Figure 4). Furthermore, the accelerated rate of wound closure in mice fed the 1% CLA supplemented diet paralleled the decrease in pI*κ*B*α* protein levels. It is well recognized that the activation of NF*κ*B is redox sensitive and can be inhibited by antioxidants [42]. It was suggested that CLA is an influential antioxidant that shows free radical scavenging activities [28]. Previous studies represented that CLA decreased NF*κ*B activation [43], confirming our data suggesting that dietary CLA supplementation may accelerate wound healing as a result of down-regulated NF*κ*B activation.

To investigate the effect of dietary CLA supplementation on NF*κ*B activation and following events during the early inflammatory stage, protein levels of the inflammatory mediator COX-2, a target gene of NF*κ*B activation, were examined at different time points. The expression of COX-2 is scarcely detectable under normal physiological states. On the contrary, it can be induced by proinflammatory cytokines, bacterial endotoxins, growth factors, and phorbol esters through NF*κ*B activation [44]. Because of its biological role in chronic inflammatory diseases [45], it is possible that down-regulated expression levels of COX-2 protein may accelerate the wound healing procedure by reducing inflammatory response. Similar to previous studies [31], we found that dietary CLA supplementation downregulated COX-2 expression. Therefore, this result suggests that dietary CLA supplementation may apply its beneficial effect via modification in the expression levels of inflammatory mediators such as COX-2 during wound healing. Because the activation of NF*κ*B is redox sensitive, the antioxidant effect of CLA blocks NF*κ*B activation and downregulates its downstream target, COX-2.

During the inflammatory phase of wound healing, innate immune cells that are present in the wound produce and secrete large amounts of ROS, which are of necessity to protect the organism against invading bacteria [11]. If ROS are produced in excessive amounts or the detoxification of ROS is insufficient, oxidative stress occurs. HO-1 is involved in the cell response against oxidative injury. The present results showed that the levels of HO-1 protein in mice fed dietary CLA supplementations were lower than those in mice fed the control diet. In addition, the levels of CuZnSOD and MnSOD protein in mice fed the dietary CLA supplementation were lower than those in mice fed the control diet. This can suggest that ROS levels be reduced in mice fed dietary CLA compared to the control and this could be the ability of CLA to quench the ROS generated by innate immune cells [28]. The controlled protein expression of several ROS-scavenging enzymes such as HO-1, CuZnSOD, and MnSOD that we observed after skin injury seems to be in contrast to findings of other researchers [46], who found enhanced expression of ROS-detoxifying enzymes at the wound site resulting in improvement of healing by regulating the levels of ROS. Oxidative stress and ROS detoxifying enzymes can always represent a double face. The up-regulation of the enzymes can help wound healing, but from the other side, it can be the consequences of an increased oxidative stress. In our previous work [35], we showed that oxidative stress can slow down the wound healing process and it was parallel to increased HO-1 levels. Therefore, our result of lowered protein expression of ROS-detoxifying enzymes in skin wound healing might be attributed to down-regulated generation of ROS confirmed in decreased MDA level in liver, possibly by dietary CLA supplementation.

## 5. Conclusions

Taken together, the results of this study suggest that dietary CLA supplementation accelerates the early inflammatory response during wound healing. Because of the critical role of NF*κ*B, which is required for the induction of an inflammatory mediator COX-2 during inflammation, reduced activation of NF*κ*B may prevent prolonged inflammatory responses by decreased oxidative stress in CLA supplementation, which is confirmed through HO-1, CuZnSOD, and MnSOD expression.

In conclusion, the current study demonstrated that CLA exerts beneficial effects on the early inflammatory response of cutaneous wound healing through the modulation of inflammatory response and oxidative stress. Therefore, the results may provide critical insight into future nutritional intervention strategies designed to enhance early wound healing not only in people with normal body weight but in overweight or obese people.

## Figures and Tables

**Table 1:** Diet composition of experimental group.

	Experimental diets*		
	Control	0.5% CLA	1% CLA
		*g/100 g*	
Casein	20	20	20
Cornstarch	39.7486	39.7486	39.7486
Dyetrose	13.2	13.2	13.2
Sucrose	10	10	10
Cellulose	5	5	5
Corn Oil	7	6.388	5.776
CLA	—	0.612	1.224
*t*-Butylhydroquinone	0.0014	0.0014	0.0014
Salt Mix #210025	3.5	3.5	3.5
Vitamin Mix #310025	1.0	1.0	1.0
L-Cystine	0.3	0.3	0.3
Choline Bitartarate	0.25	0.25	0.25

Total	100	100	100

*Control, no CLA isomers; 0.5% CLA, 0.5 g CLA/100 g diet; 1% CLA, 1 g CLA/100 g diet.

**Table 2:** Major fatty acid composition in diets.

Fatty acids	Composition %
cis-9, trans-11 CLA	37.99
trans-10, cis-12 CLA	39.09
cis-9, cis-11 CLA	3.36
trans-9, trans-11 CLA	1.22

Total CLA	81.67

**Table 3:** Relationships (Pearson's correlation coefficients) between body weight gain and wound size.

	Wound size		
	d 1 (24 hr)	d 2 (48 hr)	d 3 (72 hr)
Body weight gain	.580*	.548*	.552*

*P < .05.
